# Complexities of Occult and Obscure Gastrointestinal Bleeding: A Case Report

**DOI:** 10.7759/cureus.102391

**Published:** 2026-01-27

**Authors:** Anna Mealy, Joseph Jaya, Zacch Ray Yan Seah, Geraldine Ooi

**Affiliations:** 1 General Surgery, Monash Health, Melbourne, AUS; 2 Surgery, Monash University, Melbourne, AUS; 3 Upper Gastrointestinal Surgery, Monash Health, Melbourne, AUS

**Keywords:** anaemia, gastroenterology and endoscopy, general and colorectal surgery, general surgery, haematology, iron deficiency anaemia (ida)

## Abstract

Small-bowel vascular malformations represent an uncommon but important source of obscure bleeding that can lead to iron deficiency anemia. Diagnosis and management can be challenging due to the intermittent nature and inaccessibility of small-bowel lesions. We present a 36-year-old man with recurrent iron deficiency anemia (IDA) requiring monthly iron infusions, in whom investigations using multiple modalities revealed the unusual finding of multiple small-bowel vascular malformations. Extensive investigations have failed to definitively localize a culprit bleeding point. This case highlights the diagnostic complexity of occult small-bowel bleeding and highlights the need for a coordinated multidisciplinary approach to establish an optimal management strategy.

## Introduction

Obscure gastrointestinal bleeding (OGIB) has been defined as gastrointestinal (GI) bleeding from an unidentified origin that persists despite a comprehensive upper and lower GI evaluation [1]. Small-bowel vascular lesions such as angioectasia, Dieulafoy’s lesion, and arteriovenous malformations are the most common causes of OGIB [2]. OGIB accounts for 5%-10% of all GI bleeding incidents; however, there is no clear consensus on management strategies for the different causes [2-5].

While video capsule endoscopy and double-balloon enteroscopy have aided in the diagnosis of OGIB, we are still currently accepting a failure-to-diagnose rate of up to 50% [6]. Recurrence of bleeding is unfortunately common, with re-bleeding rates as high as 40%-60% in patients with angiodysplasia despite endoscopic intervention [2,7,8].

This case illustrates the diagnostic difficulty of occult and OGIB and the challenges in selecting optimal management. It follows a 36-year-old man who undergoes multiple repeated investigations over several years to identify the underlying cause of recurrent, symptomatic anemia. The eventual identification of multiple small-bowel vascular malformations posed challenges in determining the most appropriate definitive management.

## Case presentation

We present a 36-year-old man who was referred to our center in 2024 for symptomatic anemia and had required monthly intravenous iron infusions since 2017. At presentation in March 2024, there was no history of large-volume bright red blood per rectum or melena. He reported easy fatigability and lightheadedness. More recently, he experienced epigastric pain and weight loss. He denied any personal or family history of spontaneous epistaxis or easy bruising. He had no dietary restrictions and consumed meat almost daily. His hemoglobin trend over the years is shown in Table 1, alongside other laboratory investigations obtained during the clinic workup.

**Table 1:** Laboratory results at presentation to the emergency department and during clinic evaluation. Hb, hemoglobin; MCV, mean corpuscular volume; MCH, mean corpuscular hemoglobin; RDW, red cell distribution width; LDH, lactate dehydrogenase; INR, international normalized ratio; APTT, activated partial thromboplastin time

Blood analysis	2021	2022	2024	Reference range
Hb	110 g/L	84 g/L	79 g/L	130-180 g/L
MCV	72 fL	60 fL	61 fL	78-98 fL
MCH	23.1 pg	17.5 pg	18.0 pg	27-34 pg
RDW	29.3%	20.4%	22.8%	11%-15%
LDH	-	-	308 U/L	120-250 U/L
Iron	-	<2 mcmol/L	3 mcmol/L	11-32 mcmol/L
Ferritin	-	9 microg/L	4 microg/L	30-340 microg/L
INR	-	1.1	1.1	0.8-1.2
APTT	-	25 s	26 s	22-32 s
Fibrinogen	-	2.9 g/L	2.9 g/L	1.5-4 g/L

Coeliac, paroxysmal nocturnal hemoglobinuria, and pernicious anemia screens were negative. An abdominal ultrasound showed no sonographic evidence of cirrhosis or portal hypertension.

**Figure 1:**
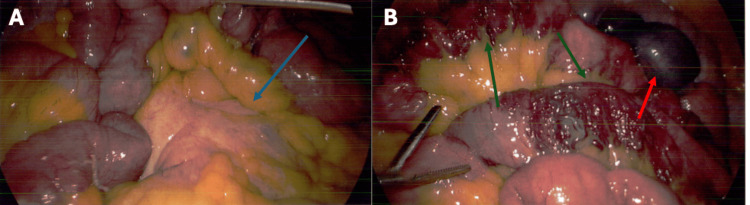
Intraoperative findings, March 2024. (A) Soft-tissue mesenteric mass (blue arrows). (B) Arteriovenous malformations concerning for angioectasia (green arrows). Red arrows highlight the tattoo on the small bowel from a previous endoscopy.

A computed tomography (CT) with intravenous contrast identified a mesenteric soft tissue mass, and a decision was made to perform a diagnostic laparoscopy and tissue biopsy. Intra-operatively, there was congestion and scarring of the mesentery (Figure 1A). Biopsies showed reactive changes only. Unexpectedly, several segments of mid-small bowel were found to have extensive ectatic vascularity over the serosa. This involved more than 1 m of bowel, from the duodenojejunal flexure to the terminal ileum (Figure 1B).

The patient had an extensive workup under a private gastroenterologist throughout the years with repeated endoscopic assessments. Gastroscopy and colonoscopy showed no convincing cause for occult blood loss. Video capsule endoscopy (VCE) from 2021 revealed multiple blue nevi and concerns for multiple arteriovenous malformations throughout the small bowel. A subsequent antegrade and retrograde double-balloon enteroscopy during the same year was normal.

**Figure 2:**
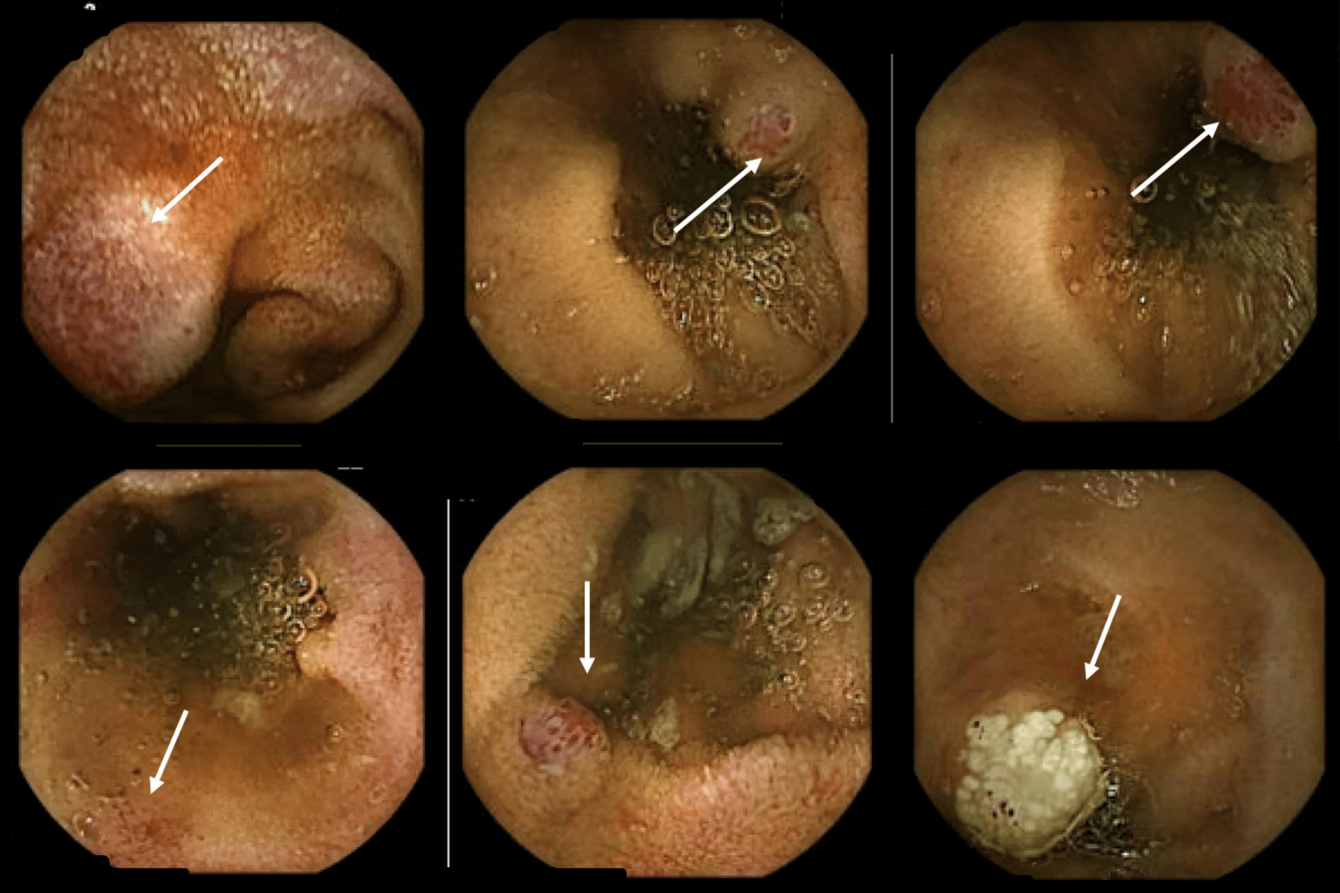
Video capsule endoscopy, July 2024. White arrows indicate abnormal small-bowel mucosa, concerning for nodularity and angioectasia. Transit time: 72%-79%.

Ongoing iron deficiency anemia prompted a repeat VCE in July 2024, which showed abnormal small bowel mucosa with some nodularity and concerns for angioectasia with active bleeding (Figure 2). 

**Figure 3:**
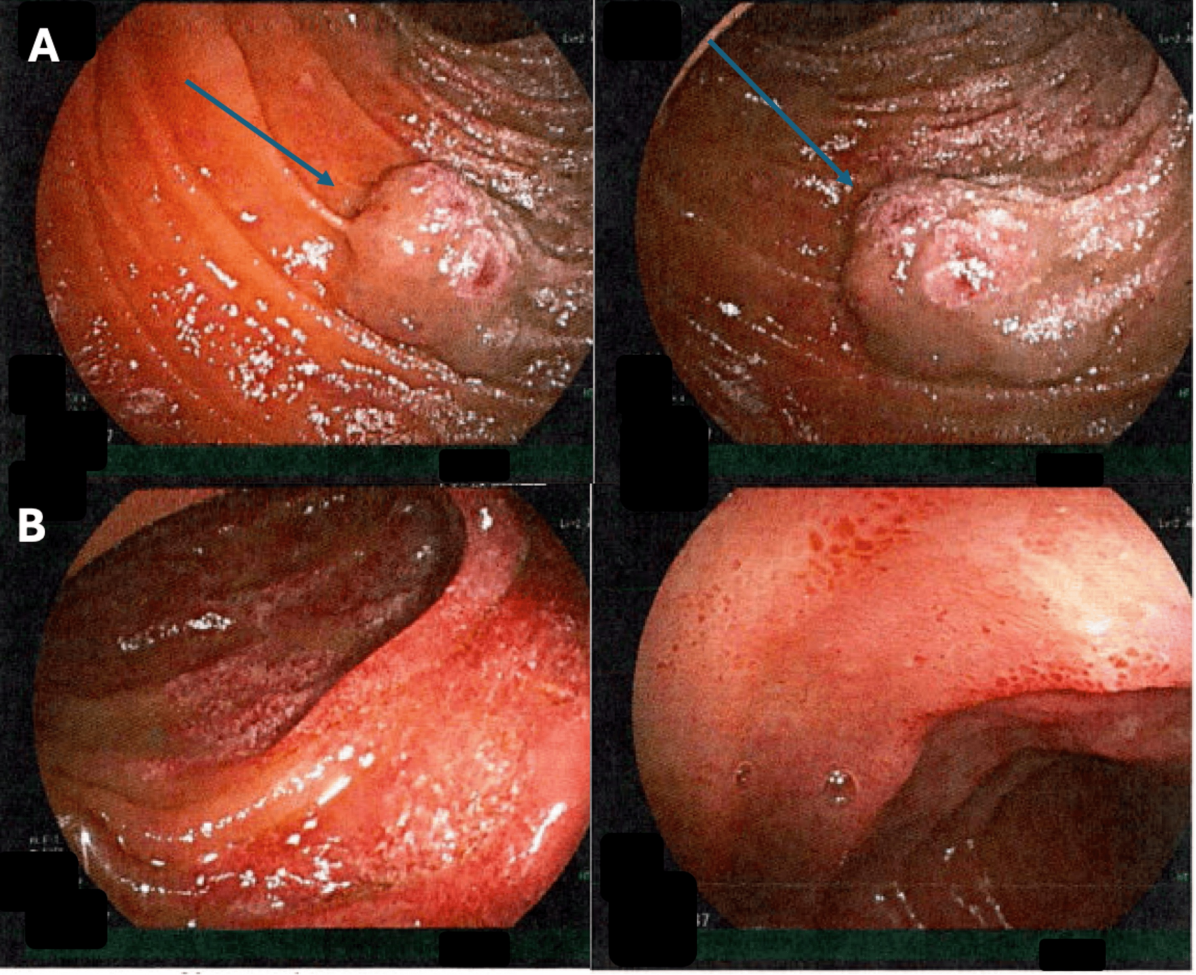
Retrograde enteroscopy, April 2025. (A) Vascular malformations in the mid-jejunum (blue arrows). (B) Surrounding abnormal mucosa in the mid-jejunum.

Antegrade enteroscopy in August 2024 to the mid-jejunum was unable to visualize these lesions. Finally, retrograde enteroscopy in April 2025 demonstrated at least three large vascular malformations in the mid-jejunum with overlying and surrounding abnormal mucosa (Figure 3). 

Discussion is ongoing with hematology, gastroenterology, and the surgical team to determine the best course of management for this patient. Options under consideration include an on-table enteroscopy with clipping, an interventional radiology approach for embolization of the vascular anomalies, or a repeat laparoscopy with targeted small-bowel resection.

## Discussion

This report highlights an interesting case of occult and OGIB and demonstrates the associated diagnostic and management challenges.

It is advised that if initial bidirectional endoscopy is negative, these investigations should be repeated [9]. Push enteroscopy can be used at this time to assess for commonly missed lesions in the distal duodenum and proximal jejunum. VCE is considered the initial diagnostic step once the upper GI tract and colon have been excluded as bleeding sources. This is typically followed by balloon-assisted or double-balloon-assisted enterostomy if required [1,10]. VCE allows for non-invasive evaluation of the entire small bowel with a diagnostic yield of 38-83% in patients with OGIB [5]. Limitations associated with capsule endoscopy include the inability to take biopsies or perform therapeutic treatments, difficulty in localizing lesions, and lack of specificity [2,11]. Double-balloon enteroscopy allows deep intubation of the small bowel using traditional endoscopes. It can be advanced 240-330 cm distal to the pylorus and 102-140 cm proximal to the ileocecal valve [11]. The diagnostic yield ranges from 60% to 80% in patients, with successful therapeutic intervention rates reported between 40% and 73% of patients [12,13]. Both of these modalities were used in this case with varying success.

Management of OGIB depends on the site and underlying etiology; however, there is little evidence from randomized controlled trials to guide optimal treatment plans [11]. Therapeutic options may include endovascular embolization, endoscopic mechanical hemostasis using clips or bands, or surgical resection [1,14]. Despite advances in endoscopic therapy, rebleeding remains common, occurring in 20%-50% of cases [11].

This case highlights the clinical dilemma posed when multiple potential bleeding sites are identified without confirmation of the definitive culprit lesion. Surgical resection is generally reserved as a last resort or for patients who have failed more minimally invasive therapy. While resection can achieve excellent outcomes in cases of discrete pathology such as tumors or localized arteriovenous malformations, diffuse lesions are far more challenging to manage [11]. In such situations, surgical resection carries significant risks, including postoperative complications such as short-bowel syndrome, and may ultimately fail to resolve the underlying problem if the true source of bleeding remains unidentified.

A combined endovascular-surgical approach may offer an alternative in complex cases such as this. This technique involves angiographic localization of small-bowel lesions preoperatively or intraoperatively to highlight the relevant vasculature and mesentery, thereby facilitating targeted intervention [2]. In cases of diffuse lesions, intraoperative enteroscopy allows for endoscopic treatment; recurrence following endoscopic management remains a recognized risk [15].

Despite extensive investigations, including multiple endoscopies and a diagnostic laparoscopy, the localization of the bleeding source remained unclear in this patient. The presence of diffuse vascular lesions complicates decision-making and highlights the importance of a multidisciplinary approach between gastroenterology, surgery, hematology, and interventional radiology in determining an appropriate management plan. This case emphasizes the need for an individualized management plan balancing diagnostic accuracy, treatment efficacy, and the potential morbidity of invasive intervention.

## Conclusions

This case highlights the diagnostic and therapeutic challenges associated with recurrent, occult GIbleeding in a young patient. Despite multiple investigations performed over several years across different healthcare systems, the underlying cause of symptomatic anemia remained elusive, resulting in cumulative procedural morbidity and significant healthcare resource utilization. The eventual identification of jejunal mucosal vascular malformations and small-bowel serosal ectasia underscored the complexity of determining an appropriate management strategy in the absence of a single, clearly targetable lesion. This case emphasizes the importance of a systematic, multidisciplinary approach to investigation and management, balancing the risks of further intervention against the potential benefits, and supports careful consideration of conservative versus definitive treatment strategies in similar presentations.
